# Adaptive evolution and demographic history contribute to the divergent population genetic structure of *Potato virus Y* between China and Japan

**DOI:** 10.1111/eva.12459

**Published:** 2017-03-02

**Authors:** Fangluan Gao, Wenchao Zou, Lianhui Xie, Jiasui Zhan

**Affiliations:** ^1^ Fujian Key Laboratory of Plant VirologyInstitute of Plant VirologyFujian Agriculture and Forestry UniversityFuzhouChina

**Keywords:** Bayesian skyline plots, demographic history, natural selection, phylogenetic analysis, *Potato virus Y*

## Abstract

*Potato virus Y* (PVY) is an important plant pathogen causing considerable economic loss to potato production. Knowledge of the population genetic structure and evolutionary biology of the pathogen, particularly at a transnational scale, is limited but vital in developing sustainable management schemes. In this study, the population genetic structure and molecular evolution of PVY were studied using 127 first protein (P1) and 137 coat protein (CP) sequences generated from isolates collected from potato in China and Japan. High genetic differentiation was found between the populations from the two countries, with higher nucleotide diversity in Japan than China in both genes and a *K*_ST_ value of .216 in the concatenated sequences of the two genes. Sequences from the two countries clustered together according to their geographic origin. Further analyses showed that spatial genetic structure in the PVY populations was likely caused by demographic dynamics of the pathogen and natural selection generated by habitat heterogeneity. Purifying selection was detected at the majority of polymorphic sites although some clade‐specific codons were under positive selection. In past decades, PVY has undergone a population expansion in China, whereas in Japan, the population size of the pathogen has remained relatively constant.

## 1 Introduction

Genetic drift, gene flow, and natural selection are three main evolutionary forces shaping the spatial population genetic structure of species (Zhan & McDonald, 2004). Under constrained gene flow, stochastic changes in allele frequencies among geographic populations can result in random fixation of neutral alleles, leading to nonadaptive differentiation (Wright, 1938). On the other hand, divergent selection for various ecological or physiological characters among genetically isolated populations may lead to adaptive population subdivision (Koskella & Vos, 2015; Yang et al., 2016). In plant pathology, understanding how populations are spatially structured is important to project the evolutionary trajectories of pathogens and formulate approaches for sustainable plant disease management (Zhan, Thrall, & Burdon, 2014). For example, many soil‐borne plant pathogens are highly spatially structured (Gilbert, 2002) and regional R gene deployment may be an appropriate method for an effective and durable control of plant disease caused by the pathogens. On the other hand, recombination can facilitate the reshufflings of genomes and R gene pyramids may be less efficient in managing sexual pathogens usually characterized by higher genetic variation distributed at a fine scale (McDonald & Linde, 2002).

Plant pathogens can vary greatly in spatial population genetic structure (Barrett, Thrall, Burdon, & Linde, 2008; Zhan, Thrall, Papaix, Xie, & Burdon, 2015), depending on the biotic and abiotic factors they associated with such as host genetics, pathogen biology, physical environments, and the ways of human intervention during and postagricultural production. These factors can affect individually and interactively on the extent of genetic drift, gene flow, and selection, therefore influencing the generation and maintenance of spatial population structure (Bergholz, Noar, & Buckley, 2011; Burdon & Thrall, 2008; Zhan & McDonald, 2013). Natural selection is expected to play a central role in the spatial population genetic structure of plant pathogens in agricultural ecosystems (Thrall et al., 2011), primarily due to variation in host genetics, fungicide applications, climatic conditions, and agricultural practices among regions. Directional selection for virulence, fungicide resistance, and other ecological characters related to particular biogeographic environments can drive the rapid accumulation of adaptive genetic differentiation in plant pathogen populations (Achtman & Wagner, 2008). At the same time though, variation in these same factors among regions can also strongly influence the demographic dynamics of plant pathogens, generating nonadaptive genetic differentiation in the pathogen populations. However, despite recognition of its importance, an in‐depth understanding of how patterns of spatial population genetic structure are generated and maintained and the main evolutionary mechanisms responsible for these patterns are still limited for many plant pathogens, but critical for sustainable plant disease management (He, Zhan, Cheng, & Xie, 2016; Zhan et al., 2015).

*Potato virus Y* (PVY) is a member of the genus *Potyvirus* in the family Potyviridae. Its genome has a single‐stranded positive‐sense RNA of ~9.7 kb, encoding a polyprotein that is cleaved into 10 mature functional proteins (King, Lefkowitz, Adams, & Carstens, 2011). Additionally, a short polypeptide (PIPO) is expressed within the P3 cistron by frame shifting (Chung, Miller, Atkins, & Firth, 2008). Among the 11 functional proteins encoded, the first protein (P1) and the coat protein (CP) are thought to play an important role in the adaptation of potyviruses to host species (Valli, López‐Moya, & García, 2007). Furthermore, the CP gene has been frequently used in strain identification, species classification, and phylogenetic analysis of potyviruses (Cuevas, Delaunay, Rupar, Jacquot, & Elena, 2012).

PVY is one of the most destructive pathogens affecting potato (*Solanum tuberosum* L.), the third largest food crop in the world (Birch et al., 2012) and widely grown in many Asian countries including China and Japan. It can cause 40%–70% yield reduction in potato production (Nolte, Whitworth, Thornton, & McIntosh, 2004) and significantly reduce the quality of seed tubers. As the largest producer in the world, China accounts for 26.3% and 22.2% of the global potato acreages and yields, respectively (Wang et al., 2011), and potato production in the country is expected to substantially increase in coming decades due to government support and dietary shifts (Kearney, 2010). PVY is one of the main factors constraining further development of Chinese potato industry (Wang et al., 2011).

Knowledge of the population genetics and evolutionary biology of PVY, particularly at a transnational scale, is relatively limited compared to other important plant pathogens such as *Magnaporthe* (Tredway, Stevenson, & Burpee, 2005), *Pyrenophthora* (Gurung, Short, & Adhikari, 2013), *Verticillium* (Short et al., 2015), and *Phytophthora* (Tian et al., 2016). Many factors such as technology and resource availability may partially contribute to this shortage. In addition, importation of living pathogens even for scientific reasons is strictly forbidden in many countries including China due to quarantine regulations. As a consequence, analysis of biotrophic pathogens with no or few morphological characters used to be very challenging for many researchers. With the advance of PCR‐based sequencing technology and sharing of sequence data in many public domains, empirical analysis of the population genetic structure and evolutionary biology of biotrophic plant pathogens such as PVY at an international scale has become possible. This approach has been used by several laboratories to infer the evolution of PVY (Cuevas et al., 2012; Ogawa, Tomitaka, Nakagawa, & Ohshima, 2008), usually involving bona fide geographical populations. Ogawa and colleagues (Ogawa, Nakagawa, Hataya, & Ohshima, 2012; Ogawa et al., 2008) compared the population structure of PVY in Japan, Europe, and North America and found some unique evolutionary patterns associated with the pathogen in Japan. For example, there were only three nonrecombinant subpopulations in Japan (PVY^O^, PVY^N^, and PVY^NTN^), whereas six subpopulations existed worldwide. Furthermore, unlike results from other regions where the subpopulations all have the same age, it is believed that the Japanese PVY^O^ subpopulation is older than the PVY^N^ and PVY^NTN^ subpopulations.

The objectives of this study were to (i) compare the population genetic structure of PVY in China and Japan and (ii) determine the main evolutionary and demographic mechanisms responsible for the observed population genetic structure.

## 2 Materials and methods

### 2.1 Viral sequences

**Figure 1:**
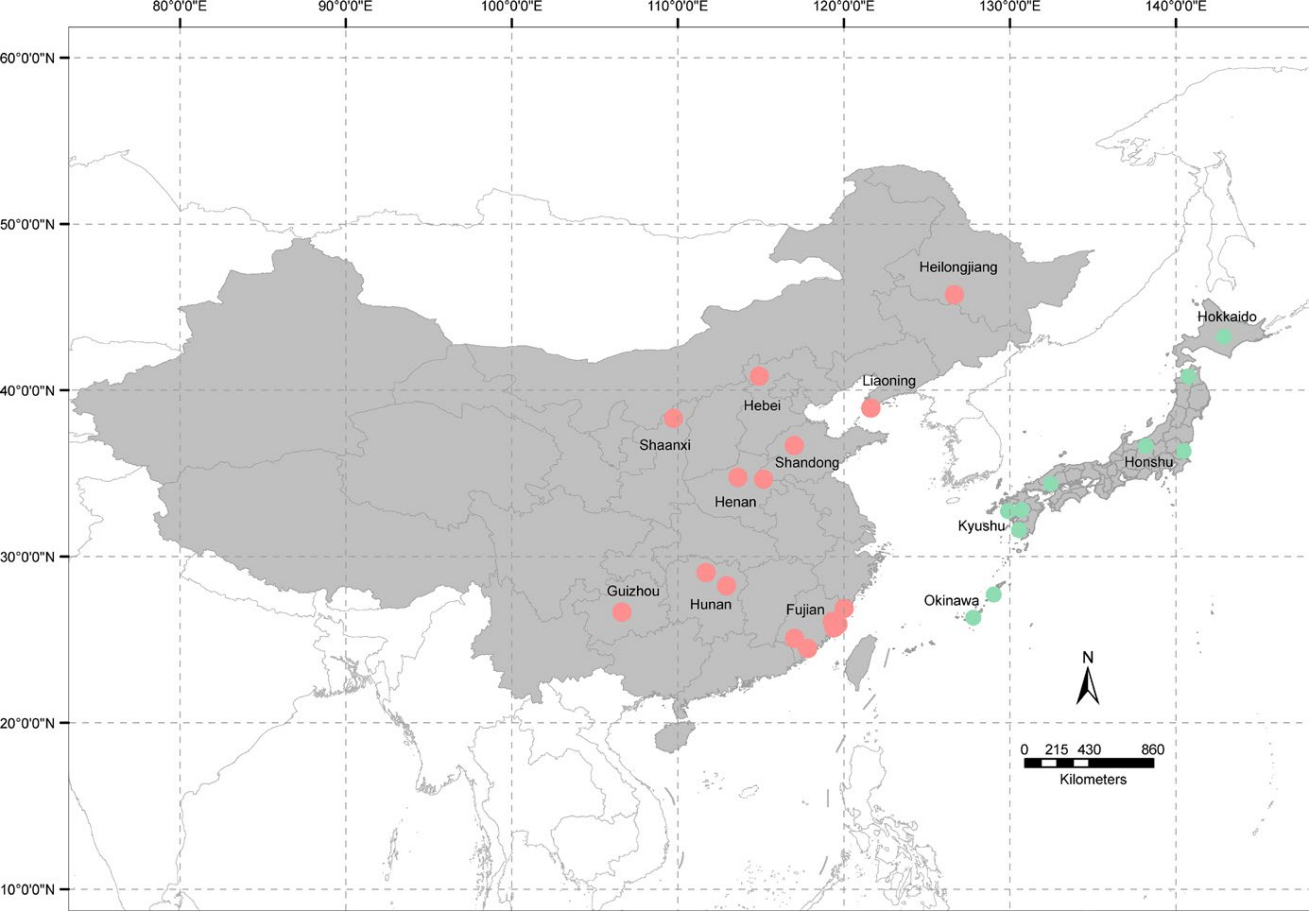
Map showing the localities of *Potato virus Y* (PVY) isolates included in this study. Arcgis 10.0 software was used to create the map. PVY isolates from China and Japan are indicated by pink and green, respectively

Eighty‐five PVY isolates, confirmed by enzyme‐linked immunosorbent assay (ELISA) with a broad‐spectrum PVY antibody (Agdia, Elkhart, USA), were randomly collected from potato (*Solanum tuberosum*) across a range of geographical locations in China including Fujian, Hunan, and Hebei provinces in 2011 and 2012 (Figure 1). Total RNAs were extracted using an RNAsimple Kit according to the manufacturer's instructions (TianGen, Beijing, China). Full‐length cDNAs were synthesized by RT‐PCR using Oligo(dt)_18_ and TransScript^®^ First‐Strand cDNA Synthesis SuperMix (TransGen, Beijing, China) and amplified with two pairs of degenerate primers as described previously (Gao et al., 2014). PCR amplifications of cDNAs were performed in a total volume of 50 μl composed of 5.0 μl of TransTaq™ 10× HiFi Buffer II, 4.0 μl of dNTPs (2.5 mM), 2.0 μl of forward primer (10 μmol/l), 2.0 μl of reverse primer (10 μmol/l), 34.5 μl of ddH_2_O, 0.5 μl of TransTaq™ HiFi Polymerase (5 U/μl), and 2.0 μl of template cDNA. The PCR program was initially denatured at 94°C for 5 min; followed by 30 cycles of 94°C for 30 s, 53°C (P1 gene), or 55°C (CP gene) for 30 s and 72°C for 1 min; ended with an extension at 72°C for 10 min. PCR products were separated on 1% agarose gels by electrophoresis, visualized using a UV transilluminator and cleaned using a TIANgel Maxi Purification Kit (TianGen).

Purified PCR fragments were ligated into T‐tailed pEASY‐T5 Zero vector (TransGen) and transformed into competent *Escherichia coli* strain Trans1‐T1 (TransGen). Recombinant plasmids were extracted and sequenced in both directions by Nanjing GenScript Biological Technology Co., Ltd. (GenScript, Nanjing, China). Due to high mutation rate in RNA viruses, at least four cDNA clones derived from two separate PCR reactions were sequenced for each PVY isolate to ensure consensus. Only the sequence identical in at least three clones was used for further analyses to eliminate potential heterogeneity introduced by Taq polymerase. All sequences generated in this study were deposited in GenBank databases. In addition, 24 sequences of P1 gene and 26 sequences of CP gene from other parts of China (including Guizhou, Shandong, Heilongjiang, Liaoning, and Shaanxi provinces) and 34 sequences each of CP and P1 genes from Japan were retrieved from GenBank (Table S1, Figure 1). Consequently, the combined sequences from China were generated from PVY samples collected between 2005 and 2012 and the Japanese sequences were generated from samples collected between 1995 and 2012. As host‐driven adaptation could affect the diversification of viral isolates, only PVY sequences derived from potato were retrieved and included in the analysis of population genetic spatial structure.

### 2.2 Genetic diversity and population genetic differentiation

Nucleotide sequences of the P1 and CP genes were aligned using the Muscle algorithm (Edgar, 2004) implemented in MEGA5 (Tamura et al., 2011). A nucleotide identity matrix was generated using BioEdit software (Hall, 1999) after all gaps were removed. Haplotype diversity (*H*_d_) and nucleotide diversity (π) were estimated using DnaSP 5.0 (Librado & Rozas, 2009). Pairwise *F*_ST_, a measure of genetic differentiation among populations, was computed in Arlequin 3.5 (Excoffier & Lischer, 2010). Genetic differentiation among populations was also evaluated by *K*_ST_ and *S*_nn_ using DnaSP 5.10 (Hudson, 2000; Hudson, Boos, & Kaplan, 1992; Librado & Rozas, 2009). The hypothesis of deviation from null population differentiation was tested by 1000 permutations of the original data.

### 2.3 Recombination and phylogeography analyses

Putative recombination joints (RJ) and parental sequences were identified using seven methods (RDP, GENECONV, BOOTSCAN, MaxChi, CHIMAERA, SiSCAN, and 3SEQ) implemented in the RDP4 suites (Martin et al., 2010). The probability of a putative recombination event was corrected by a Bonferroni procedure with a cutoff of *p *<* *.01. To avoid false identification, only events supported by at least four of the seven methods were considered to be recombinants. Recombinants were removed from the subsequent reconstruction of phylogenetic trees.

Phylogenetic trees were reconstructed by the Bayesian inference (BI) implemented in MrBayes 3.2.5 (Ronquist et al., 2012) and maximum likelihood (ML) implemented in MEGA5 (Tamura et al., 2011) using the *GTR + G + I* nucleotide substitution model determined by MrModeltest (Nylander, 2008). BI was run in 2,000,000 generations of Markov chains that were sampled every 100 generations to establish convergence of all parameters. The effective sample size (ESS) of parameters was checked by Tracer 1.6 to ensure values above 200 as advised by the programmers with the first 25% of sampled trees burn‐in. Topology robustness of ML trees was assessed by 1,000 bootstraps. ML‐BPs and BI‐PPs were plotted on Bayesian 50% majority‐rule consensus trees using FigTree 1.4.2 and Illustrator CS5 (Adobe).

The effect of geographic origin on PVY populations was evaluated by phylogeny–trait association analysis, and BaTS 2.0 (Parker, Rambaut, & Pybus, 2008) was used to compute an association index (AI), parsimony score (PS), and maximum monophyletic clade (MC). Low AI and PS scores and high MC scores suggest a strong PVY–geography association. The topology of reconstructed trees was tested using BaTS by comparing it with the randomized trees generated from 10,000 reshufflings of tip characters. Topology robustness was determined in BaTS by comparing it with the null distribution of trees obtained from 10,000 bootstraps of tip characters.

### 2.4 Natural selection

HyPhy 2.10b (Kosakovsky Pond, Frost, & Muse, 2005) and PAML 4.7 (Yang, 2007) were used to identify nucleotide sites in P1 and CP cistrons that were likely to be involved in PVY adaptation. Three codon‐based approaches, that is, IFEL (internal branches fixed‐effects likelihood), REL (random‐effects likelihood), and MEME (mixed effects model of evolution) (Kosakovsky Pond et al., 2006, 2011; Murrell et al., 2012), were included in the HyPhy package. Only sites simultaneously identified by IFEL and MEME with *p *<* *.05 and >.95 posterior probability identified by REL were considered to be under selection. In addition, the ratio of nonsynonymous (dN) to synonymous (dS) substitution (ω = dN/dS) was calculated for each gene using CODEML algorithm implemented in PAML 4.7 (Yang, 2007). Three different nested models (M3 vs. M0, M2a vs. M1a, and M8 vs. M7) were compared and likelihood‐ratio tests (LRTs) were applied to select the one that best fitted the data. When the LRT was significant (*p‐*value < .01), the Bayes empirical Bayes (BEB) method (Yang, Wong, & Nielsen, 2005) was used to identify amino acid residues that are likely to have evolved under positive selection based on a posterior probability threshold of .95.

### 2.5 Population historic dynamics

Two different approaches were used to explore the demographic history of PVY populations in the two countries. First, Tajima's *D* and Fu's *F*_S_ statistics implemented in Arlequin 3.5 (Excoffier & Lischer, 2010) were used to determine the neutrality of the P1 and CP genes by 1,000 permutations of the original sequences. Tajima's *D* test identifies evolutionary events such as population expansion, bottlenecks, and selection by comparing the estimated number of segregating sites with the mean pairwise difference among sequences (Tajima, 1989). Fu's *F*_S_ is sensitive to population demographic expansion and usually displays a negative value (Fu, 1997) in expanding populations. Following these calculations, Bayesian skyline plots (BSP) was generated to explore demographical history using BEAST 1.8.2 (Drummond, Suchard, Xie, & Rambaut, 2012). Sampling times of the sequences were used to calibrate the molecular clock. Date‐randomization tests (DRTs) were performed in R 3.3.1 using the Tip Dating Beast package (Rieux & Khatchikian, 2016) to determine the temporal signal in data sets. A data set was considered to have an adequate spread in sampling time if its average rate did not fall within the 95% confidence intervals (CIs) generated from 20 replicates of randomized dates (Ramsden, Holmes, & Charleston, 2009; Duchêne, Duchêne, Holmes, & Ho, 2015). A Bayes factors test indicated that the relaxed uncorrelated exponential model was a better fit to the sequence data than the relaxed uncorrelated lognormal model and was chosen to estimate the molecular clock of P1 and CP genes. The MCMC was run for 5 × 10^8^ generations to ensure convergence of all parameters. Convergence and ESS (>200) of the parameters were checked using Tracer 1.6.

## 3 Results

### 3.1 Sequence variation in P1 and CP genes

Seventy‐three P1 and 78 CP genes were sequenced in this study and were deposited in GenBank (Table S1). Two P1 sequences had a nucleotide T insertion at position 33 (accession number: KF722821) and a nucleotide (A) insertion at position 814 (accession number: KF771018), respectively. Moreover, two P1 sequences (accession numbers: KX451346 and KX451344) contained premature termination codons (PTCs) at position 208–210 and 619–621. A single CP sequence (accession number: KC296822) had an 11‐nucleotide deletion at the beginning of the gene. These nonfunctional sequences were removed from further analyses of the population genetic structure. In addition, 34 complete P1 and CP sequences originated from Japan were retrieved from GenBank (Table S1). Consequently, a total of 127 complete P1 and 137 complete CP sequences were included in the population genetic analysis of the virus.

The average nucleotide identities in the P1 and CP genes among the viral isolates from China were 89.9% (72.3%−100%) and 96.3% (87.0%−100%), while those from Japan were 89.1% (71.5%−100%) and 95.7% (88.7%−100%), respectively. All typical motifs of potyviruses were detected in the deduced amino acid sequences. However, the conserved N^25^ of CP protein in the reference sequences (O‐139, N‐605, C1‐SON41, Adgen, and Chile3) was replaced by S (isolates NTNHIR3 and T13) or T (isolate ONGOB6) in the Japanese isolates and the E^68^ residue was replaced by G (isolates XQ03) or K (isolate Laiwu3) in Chinese isolates (Figure S1).

One hundred and seventeen haplotypes were identified in the 127 complete sequences of P1 gene with an overall haplotype diversity of .998 and nucleotide diversity of .119 when the sequences from China and Japan were considered together (Table 1). The most common haplotype was detected four times in P1 sequences and 10 times in CP sequences. No identical haplotypes were detected in the two countries in both genes. When the sequences were considered according to individual geographic location, higher nucleotide but lower haplotype diversity was found in Japan than in China. In the CP genes, 121 haplotypes were identified in the 137 complete sequences, with an overall haplotype diversity of .984 and nucleotide diversity of .053 when sequences from the two countries were pooled. Similar to the P1 gene, higher nucleotide diversity but lower haplotype diversity was found in the CP gene from Japan than from China when they were considered separately.

**Table 1:** Sample sizes and genetic variation of the P1 and CP genes in the *Potato virus Y* populations sampled from China and Japan

Gene	Country	Sample size	Haplotypes	Haplotype diversity	Nucleotide diversity
P1	China	93	90	.999	.100
	Japan	34	27	.980	.109
	All	127	117	.998	.119
CP	China	103	99	.999	.036
	Japan	34	22	.914	.043
	All	137	121	.994	.053

### 3.2 Genetic differentiation between sequences from China and Japan

*K*_ST_, *S*_nn_, and *F*_ST_ were .141, .972, and .294 in the P1 gene and .289, .949, and .520 in the CP gene (Table 2), respectively. All of these indexes were significantly higher than the theoretical expectation of no population differentiation. *K*_ST_ and *F*_ST_ values were higher in the CP gene than in the P1 gene.

**Table 2:** Statistical tests for population differentiation between P1 and CP genes in the *Potato virus Y* populations sampled from China and Japan

Gene	*K*_ST_	*p*	*S*_nn_	*p*	*F*_ST_	*p*
P1	.141	.000***	.972	.000***	.294	.000***
CP	.289	.000***	.949	.000***	.520	.000***
Concatenated	.216	.000***	.977	.000***	.390	.000***

^*^.01 < *P *< .05.

^**^.001 < *p *< .01.

^***^ *P *< .001.

### 3.3 Recombination analyses

Forty‐two P1 sequences, all originating from China, were identified with a high level of confidence as recombinants by all seven methods implemented in the RDP package (*p*‐values ≤1.27 × 10^−2^, Table S2). All of these sequences were derived from three recombination events. Similarly, three recombination breakpoints were detected in eight CP sequences from China and four CP sequences from Japan by at least six of the seven approaches (*p*‐values ≤1.69 × 10^−2^, Table S2).

### 3.4 Phylogenetic analysis

**Figure 2:**
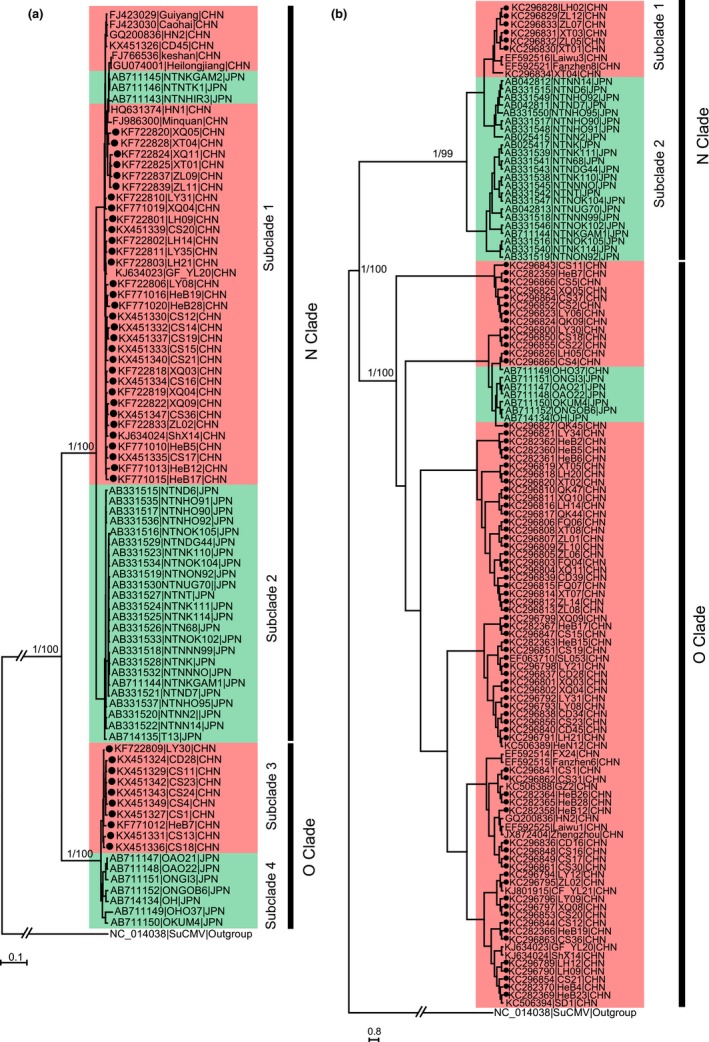
Bayesian phylogenetic trees of *Potato virus Y* (PVY) isolates based on P1 (a) and CP (b) coding regions. For three key nodes in a tree, the Bayesian posterior probabilities and maximum likelihood bootstrap percentage are indicated above the branches (Bayesian posterior/bootstrap). PVY isolates from this study are indicated by black dots. The distance unit is substitutions/site

After removal of putative recombinants, 85 complete P1 sequences and 125 complete CP sequences were included in the phylogenetic analysis. Both P1 and CP sequences fell into two distinct clades (N clade and O clade) with high posterior probabilities (PPs ≥ .99) and maximum likelihood (ML) bootstrap (BPs ≥ 70%) supports (Figure 2). In the N clade of P1 tree, all 41 Chinese isolates were clustered into subclade 1 and all but three (NTNHIR3, NTNKGAM2, and NTNTK1) of the 27 Japanese sequences were grouped into subclade 2. Similarly, all 10 Chinese isolates were clustered into subclade 3 and all seven Japanese isolates were grouped into subclade 4. In the N clade of the CP tree, all nine Chinese sequences were clustered into subclade 1, whereas all 23 Japanese sequences were grouped into subclade 2. In contrast, O clade was composed of a mixture of Chinese and Japanese sequences. A similar pattern was found when only the P1 and CP sequences from GenBank (i.e., excluding the sequences generated in this study) were used to construct a phylogenetic tree (data not shown). Distinct differences in the population genetic structure of PVY between China and Japan were also found by phylogeny–geography association analysis. Significant MC (*p* < .01), AI (*p* < .001), and PS (*p* < .001) were detected in both P1 and CP genes when sequences from the two countries were compared (Table 3). However, in general, a phylogeny–geography association was not found within countries (MC: *p* > .05; Table 3) with the exception of PVY isolates from Fujian and Heibei in China and Kyushu and Honshu in Japan.

**Table 3:** Tests of geography–phylogeny association for P1 and CP genes in PVY isolates originated from China and Japan

Gene	Country	Statistic	Isolates	Observed mean (95% HPD)	Null mean (95% HPD)	Significance
P1		AI		0.06 (0.02, 0.19)	4.61 (3.72, 5.35)	<.001***
		PS		3.15 (3.00, 4.00)	26.79 (23.77, 29.41)	<.001***
	China		51	30.71 (28.00, 31.00)	4.75 (3.31, 7.16)	.01**
		MC (Guizhou)	2	1.44 (1.00, 2.00)	1.01 (1.00, 1.05)	1.00^ns^
		MC (Heilongjiang)	2	2.00 (2.00. 2.00)	1.01 (1.00, 1.03)	.02*
		MC (Henan)	1	n/a	n/a	n/a
		MC (Hunan)	20	6.00 (6.00, 6.00)	1.98 (1.46, 2.54)	.02*
		MC (Fujian)	17	5.80 (4.00, 6.00)	1.78 (1.33, 2.27)	.01**
		MC (Hebei)	7	3.91 (2.00, 4.00)	1.16 (1.00, 1.75)	.01**
		MC (Shaanxi)	2	1.00 (1.00, 1.00)	1.00 (1.00, 1.03)	1.00^ns^
	Japan		34	23.42 (17.00, 24.00)	2.82 (2.22, 3.82)	.01**
		MC (Kyushu)	18	3.58 (2.00, 6.00)	1.88 (1.32, 2.84)	.05*
		MC (Okinawa)	3	1.14 (1.00, 2.00)	1.04 (1.00, 1.15)	1.00^ns^
		MC (Hokkaido)	7	2.61 (1.00, 4.00)	1.18 (1.00, 2.00)	.07^ns^
		MC (Honshu)	6	2.00 (2.00, 6.00)	1.09 (1.00, 1.30)	.02*
CP		AI		0.14 (0.08, 0.35)	4.90 (4.10, 6.65)	<.001***
		PS		3.85 (3.00, 4.00)	27.13 (24.76, 28.65)	<.001***
	China		95	74.29 (55.00, 82.00)	8.49 (6.18, 11.33)	.01**
		MC (Guizhou)	2	1.00 (1.00, 1.00)	1.00 (1.00, 1.00)	1.00^ns^
		MC (Shandong)	7	1.65 (1.00, 2.00)	1.11 (1.00, 1.55)	.03*
		MC (Henan)	2	1.00 (1.00, 1.00)	1.00 (1.00, 1.00)	1.00^ns^
		MC (Hunan)	24	2.48 (2.00, 4.00)	1.98 (1.55, 2.41)	.43^ns^
		MC (Fujian)	45	7.23 (7.00, 9.00)	2.87 (2.21, 3.66)	.01**
		MC (Hebei)	12	2.19 (2.00, 3.00)	1.27 (1.01, 1.99)	.05*
		MC (Shaanxi)	3	1.40 (1.00, 2.00)	1.01 (1.00, 1.04)	1.00^ns^
	Japan		30	22.83 (22.00, 23.00)	2.19 (1.75, 3.05)	.01**
		MC (Kyshu)	17	3.11 (2.00, 5.00)	1.58 (1.14, 2.13)	.02*
		MC (Okinawa)	3	1.12 (1.00, 2.00)	1.01 (1.00, 1.04)	1.00^ns^
		MC (Hokkaido)	6	2.26 (2.00, 3.00)	1.05 (1.00, 1.14)	1.00^ns^
		MC (Honshu)	4	2.00 (2.00, 2.00)	1.02 (1.00, 1.12)	.02*

AI, association index; PS, parsimony score; MC, maximum monophyletic clade; HPD, highest probability density interval; n/a: no data available because of insufficient sample size (*n *<* *2).

Significance thresholds: *.01 < *p *<* *.05; **.001 < *p *<* *.01; *** *p *<* *.001.

### 3.5 Detecting natural selection

**Figure 3:**
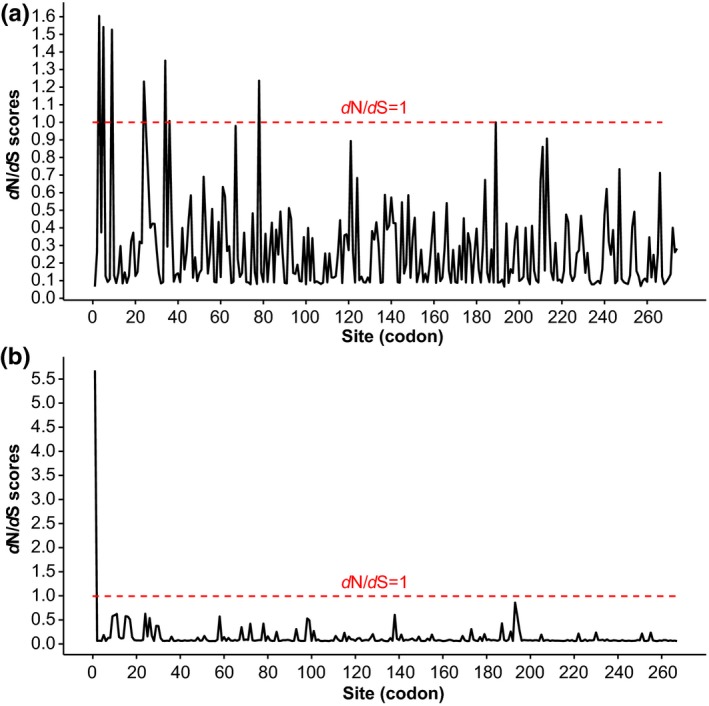
Sliding window plot of dN/dS ratios for P1 (a) and CP (b) genes. Sites under neutral (dN/dS = 1) are indicated in red dotted line. The window size is 20 codons, and the offset between windows is one codon

Purifying selection was detected in the majority of polymorphic sites by PAML packages (Figure 3). Evidence of positive selection was detected in the 1st codon of CP sequences in the O clade using the PAML approach (PP > .99, Table 4). No positive selection was detected in the N clade. In P1 sequences, no positive selection was detected in either the N or O clade by PAML (Table 4). However, positive selection in the P1 gene was detected in the 3rd and 5th codons of the N clade and the 3rd, 78th, and 241st codons of the O clade by the REL approach implemented in the HyPhy package.

**Table 4:** Putative codons in the P1 and CP sequences detected under positive selection using the PAML package and three approaches implemented in the HyPhy Software

Gene	Clade	PAML		HyPhy					
		Model M8^a^		IFEL		REL		MEME	
		Codon	PP^b^	Codon	*p* Value	Codon	PP^b^	Codon	*p* Value
P1	N Clade					3	.999^c^		
						5	1.000^d^		
	O Clade					3	.970^c^		
						78	.984^c^		
						241	.972^c^		
CP	N Clade					1	.994^d^	1	.029^c^
	O Clade	1	.999^c^	1	.023^c^	1	.991^d^	1	.007^d^
				138	.042^c^	9	.981^c^		
						11	.959^c^		
						15	.977^c^		
						58	.982^c^		

a For each PVY clade, model M8 performed better than model M7 in an LRT. Model M2a also performed better than models M0, M1a, or M3 in LRTs and provided results similar to M8.

b PP, Posterior probability that individual codon positions belong to the positively selected category using the Bayes Empirical Bayes (BEB) method implemented in PAML and the random‐effects likelihood (REL) approach implemented in the HyPhy, respectively.

c Posterior probability >95% or *p *< .05.

d Posterior probability >99% or .001 < *p *< .01.

The REL approach also detected positive selection in the 1st, 9th, 11th, 15th, and 58th codons of CP sequences in the O clade and in the 1st codon of the N clade. Similarly, positive selection was found in the 1st codon both in the O and N clades of CP sequences by the MEME approach (*p* < .05; Table 4). Positive selection was also found in the 1st and 138th codons of CP sequences in the O clade by IFEL (<.05; Table 4). However, only the 1st codon in the N‐terminal region of the CP protein, one of the cleavage sites in the PVY genome, was found to be under positive selection by both PAML and three approaches implemented in the HyPhy package. Further analysis showed that Glu (87.50%) dominated at the NIb/CP cleavage site in the N clade of the CP protein, whereas Ala (40.86%) and Glu (56.99%) were found to have a high frequency at the cleavage site in the O clade of the CP protein.

### 3.6 Demographic history

**Figure 4:**
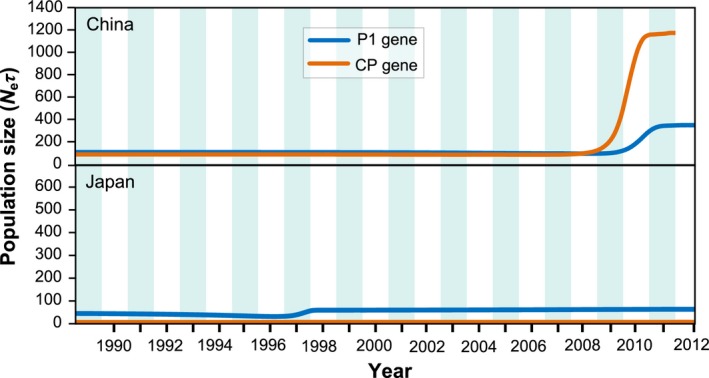
Population dynamics of genetic diversity in *Potato virus Y* (PVY). Bayesian skyline plots of the P1 and CP segments for PVY in China (top) and Japan (bottom). The *y*‐axes represent a measure of relative genetic diversity, and *x*‐axis is measured in calendar years

Significantly negative Fu's *F*_S_ was detected in both P1 and CP sequences from China (Table 5); Tajima's *D* was positive for the P1 sequences and negative for the CP sequences from China, but none of them were significant. All Tajima's *D* and Fu's *F*_S_ in the sequences from Japan were positive but not significant (Table 5). All data included in the demographic analysis passed the DRTs that showed no overlaps between the original estimate of evolutionary rate and 95% CIs generated from 20 replicates of date randomization (Figure S2). Coalescence‐based BSP revealed an explicit demographic history for the PVY populations of China and Japan (Figure 4)—showing that the PVY from China experienced a population expansion prior to a period of stability, whereas the population from Japan remained relatively constant throughout the past decades.

**Table 5:** Neutrality tests for P1 and CP sequences of PVY originated from China and Japan by Tajima's *D* and Fu's *F*_S_

Population	P1		CP	
	Tajima's *D*	Fu's *F*_S_	Tajima's *D*	Fu's *F*_S_
China	.649^ns^	−10.605**	−1.285^ns^	−23.966***
Japan	.771^ns^	2.050^ns^	.702^ns^	2.387^ns^

^*^.01 < *p *< .05.

^**^.001 < *p *< .01.

^**^ *p *< .001.

## 4 Discussion

Consistent with previous results (Ogawa et al., 2008, 2012; Tian et al., 2011), high genetic diversities (Table 1) were found in both P1 and CP genes in the current study. Each of these two genes encodes a protein with ecological functions that are important to the survival and adaptation of PVY in local biotic and abiotic environments (Quenouille, Vassilakos, & Moury, 2013). In addition to the high mutation rate in RNA viruses (Domingo, Sheldon, & Perales, 2012), high genetic variation found in both current and previous studies is possibly attributed to the high recombination rate in PVY populations (Gibbs & Ohshima, 2010). In recent decades, recombinant PVYs were found to be prevalent in the global population of the pathogen sampled from potato production areas (Quenouille et al., 2013). In this study, 52.69% and 11.76% of concatenated sequences in the Chinese and Japanese populations show a mixed genomic structure of PVY^N^ and PVY^O^ (Table S2). These results re‐enforce widespread concern related to the propensity for rapid adaptation of PVY to changing biotic and abiotic environments. Such adaptation may cause problems for the deployment of major gene‐mediated host resistance in potatoes, tomato, and other crops (Karasev & Gray, 2013).

Our study also reveals a significant difference in the molecular population genetic structure between the PVY sequences originating from potato hosts in China and Japan—a pattern that is supported by comparative analyses of genetic variation (Table 1), population differentiation (Table 2), and phylogeny–geography association (Table 3, Figure 2) in the two genes (each represented by ~130 sequences). It has been documented that measurements of the diversity of genetic variation are highly sensitive to sample sizes (Bashalkhanov, Pandey, & Rajora, 2009) and populations with larger sample sizes tend to have higher observed genetic diversity. Despite smaller sample sizes included in the Japanese than Chinese populations, both P1 and CP sequences originating from Japan show higher nucleotide diversity than those from China, suggesting that differences in nucleotide diversity between the two viral populations are unlikely to be the result of sampling error. However, the higher haplotype diversity in Chinese sequences may be associated with the somewhat larger sample size as compared to Japanese sequences (93 vs. 34 isolates). Indeed, when we used a bootstrapping approach described previously (Zhan, Pettway, & McDonald, 2003) to standardize sample sizes with the smaller population (Japan in this case) and recalculated haplotype diversity, the difference in genetic variation between the two populations disappeared (Table S3). The bootstrap was conducted using the Resampling Stats add‐in package for Excel (Blank, Seiter, & Bruce, 2001) with 100 replicates. For each bootstrap replication, haplotype diversity was recorded from a random sample of 34 sequences (the actual size of Japanese population). The mean and variance of the diversity were calculated and used for a *t*‐test.

Three indices were used to test population differentiation between Chinese and Japanese PVY sequences. *K*_ST_ and *F*_ST_ measure the relative proportion of total genetic diversity attributable to among‐population differences. They range from .00 to 1.00. A value of one for *K*_ST_ or *F*_ST_ indicates that populations investigated are completely isolated, while a value of zero indicates the populations studied are identical (Hudson et al., 1992). *K*_ST_ or *F*_ST_ values between .15 and .25 indicate high population differentiation, and values greater than .25 indicate very high genetic differentiation among populations (Balloux & Lugon‐Moulin, 2002). *S*_nn_ measures how often the nearest sequences are found in the same populations. Its value would be close to one when populations are highly structured and near .5 when populations are identical (Hudson, 2000). Therefore, *K*_ST_ and *F*_ST_ are approximately equal to *S*_nn_—.5. All three indices are higher than .15 in the current study (Table 2), suggesting a high differentiation of PVY sequences between the two populations. *K*_ST_ is the most powerful index for detecting spatial population structure for recombination and high mutation populations (Hudson et al., 1992), which is the case in PVY (Blanchard, Rolland, Lacroix, Kerlan, & Jacquot, 2008).

Phylogenetic analyses provide additional support for differentiation between the Chinese and Japanese populations of PVY (Figure 2). Sequences tend to cluster according to their geographic origin, consistent with previous results derived from more and wider geographic locations (Cuevas et al., 2012). Although some clades such as the O clade of the CP tree are composed of sequences from both locations, the hypothesis of random association between PVY sequences from China and Japan and their geographic origins is rejected by all three statistics (AS, MC and PS) (Table 3). Phylogenetic trees are reconstructed under the assumption of constant evolutionary rates and many evolutionary processes may affect the correct reconstruction of phylogenetic topology (Revell, Harmon, & Collar, 2008). The polyphyletic nature of these different clades could be generated by some sequences experiencing a complex evolutionary history such as recombination. For example, in the P1 tree, three Japanese sequences (NTNHIR3, NTNTK1, and NTNKGAM2) were clustered together with Chinese sequences in a subclade (Figure 2). It is likely they are recombinants that were not detected by the techniques used here. Our analyses suggest that the CP sequences of these three isolates are also most likely derived from recombination (Table S2).

Both stochastic and deterministic events could lead to the observed pattern of spatial structure (Caruso et al., 2011). Stochastic events caused by genetic drift may lead to nonadaptive population differentiation between the two countries. In nature, the extent of population differentiation generated by random genetic drift is expected to increase over geographic distance. Although geographic distance between some locations (ex. 2,771.10 km between Guizhou and Heilongjiang) within China is greater than that between China and Japan (ex. 819.41 km between Fujian in China and Okinawa in Japan), PVY sequences were clustered according to country origin (Figure 2, Table 3). Because our data (non‐neutrality) are not appropriate for testing the stochastic event, the contribution of genetic drift could not be ruled out. However, we believe that deterministic events are the main factor responsible for the observed pattern of spatial population genetic structure in the pathogen.

The hypothesized contribution of deterministic events to the observed pattern of spatial population genetic structure is supported by neutrality tests in the deduced amino acid sequences and in the sequence–geography association analysis. The finding that most codons in the P1 and CP sequences were under purifying selection suggests that most mutations in the PVY genomes are harmful and consequently eliminated by natural selection. In this case, the selective agents may be habitat differences between the two countries such as differences in the potato cultivars grown and climatic conditions. The main potato cultivars grown in Japan include Irish Cobbler, May Queen, Kitaakari, Touya, Dejima, Nishiyutaka, Toyoshiro, Konafubuki, Northern Ruby, and Shadow Queen (Kawakami, Oohori, & Tajima, 2015). None of these cultivars are used in China. In China, potatoes are grown over a much wider geographic area, ranging from a subtropical climatic zone in the south (e.g., Fujian) to a temperate continental climate zone in Northern China (e.g., Heilongjiang) using more diverse cultivars (Jansky, Jin, Xie, Xie, & Spooner, 2009), while in Japan, potatoes are mainly grown in the north (Kawakami et al., 2015).

Interestingly, the 1st codon translated to the cleavage site in the CP protein was detected to be under positive selection by both PAML and HyPhy with high confidence levels (PP > .99 or *p* < .05, Table 4) and the signal of positive selection increased when more sequences were included in the analysis (data not shown). Positive selection in cleavage sites has also been found in other viruses such as the human immunodeficiency virus (HIV, Banke, Lillemark, Gerstoft, Obel, & Jørgensen, 2009). Successful cleavages to form functional cis‐elements are crucial for survival and reproduction of PVY (Tena Fernandez et al., 2013). This process is catalyzed by proteases that are under constant change by mutation (Yu, Benton, Bovee, Sessions, & Lloyd, 1995). Positive selection in the CP cleavage site could serve as a reversal mechanism compensatory to mutations in the protease in PVY and other viruses (Banke et al., 2009).

Differences in spatial structure between the Chinese and Japanese sequences may also result from differences in the demographic dynamics of the PVY populations. Sudden increases or decreases in population size associated with demographic events can affect the generation, maintenance, and distribution of genetic variation, not only directly through genetic drift and mutation, but also indirectly through impacts on the efficiency of natural selection to remove or amplify mutations as well as on migration and recombination (Wang & Whitlock, 2003). Indeed, when we performed demographic analyses, we found that PVY populations in China were small but had undergone recent expansion, possibly associated with increasing potato production in the current years (Wang et al., 2011), while in Japan, they were large but stably maintained (Figure 4). This result is consistent with the potato cultivation of the two countries in the past decades. Potato acreage in Japan maintained stable over the last several decades (Kawakami et al., 2015) while increased in China from ~2.3 million hectares in 1980s to ~5.4 million hectares recently (http://faostat.fao.org). The temporal scale of samples can impact the estimate of demographic dynamics (Drummond, Pybus, & Rambaut, 2003). In our study, the temporal scale (1995–2012) in the PVY sequences from Japan was 10 years longer than that (2005–2012) from China. However, we do not believe that this difference would have affected our conclusions because all data passed the DRTs (Ramsden, Holmes & Charleston, 2009; Duchêne et al., 2015) with a high level of confidence.

In summary, our study represents one of a few attempts to understand patterns and causes of spatial population genetic structure across political borders in PVY, a destructive pathogen of potato and many other *Solanaceous* crops. The finding of two subpopulations indicates distinct gene pools exist in PVY from China and Japan. This suggests that strict quarantine regulation is needed to prevent the movement of novel alleles or allelic combination of PVY between the two countries when trading plant materials that are hosts for this pathogen (e.g., potato and tobacco). However, sequences included in this analysis were relatively limited both in sample sizes and sites. In particular, the temporal scales might be relatively short for analysis of demographic events. Further study with larger, multiple‐location, and longer temporal scale samples may be required to confirm the results and generalize the findings.

## Data archiving statement

Sequence data obtained in this study are available on GenBank under the accession numbers KF722798–KF722799, KF722801, KF722804–KF722805, KF722807–KF722808, KF722810, KF722812–KF722813, KF722815–KF722818, KF722820–KF722825, KF722827, KF722829–KF722830, KF722832, KF722834–KF722841, KF771008–KF771016, KF771018–KF771020, KX451323–KX451351 (P1 gene), and KC282358–KC282367, KC282369–KC282370, KC296789–KC296790, KC296792, KC296794, KC296796–KC296799, KC296801, KC296803–KC296807, KC296809–KC296815, KC296819–KC296825, KC296827–KC296833, KC296835–KC296859, and KC296861–KC296866 (CP gene).
