# Supplementary figures and images for: Adaptive evolution and demographic history contribute to the divergent population genetic structure of Potato virus Y between China and Japan
Source: Evol Appl. 2017 Mar 2;10(4):379–90. doi: 10.1111/eva.12459 (PMC5367074; doi:10.1111/eva.12459)

Figure S1

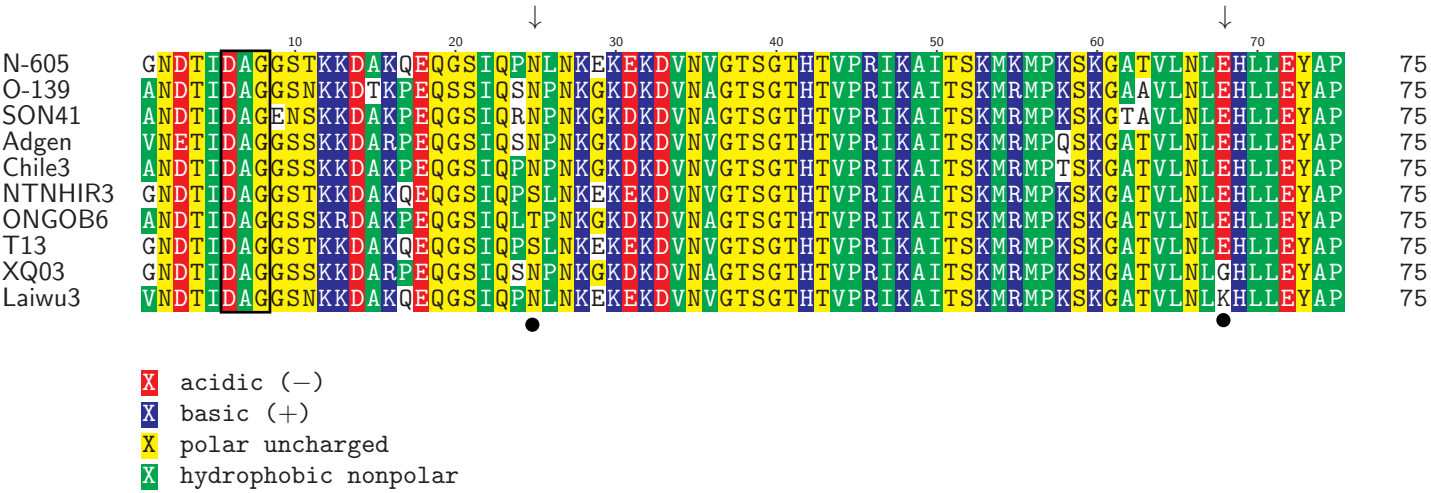

Supplement: Supplementary file 1 [file EVA-10-379-s001.pdf]

Figure S2

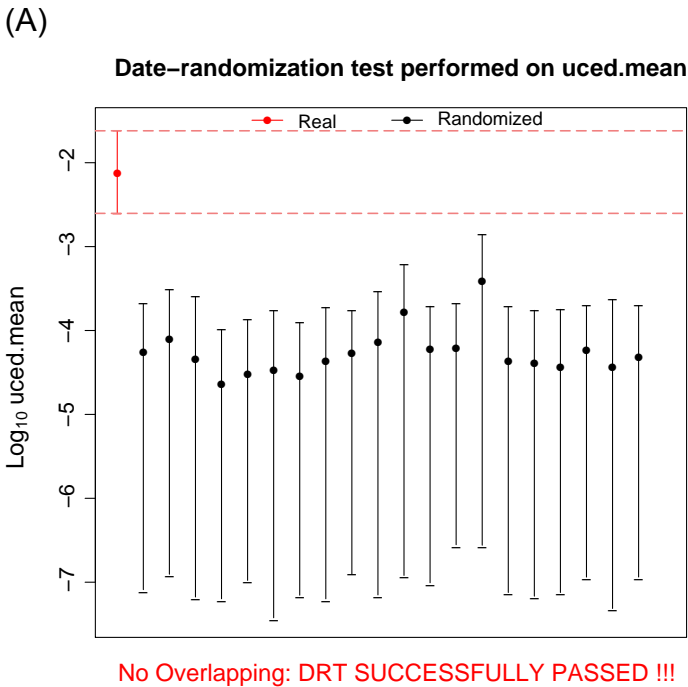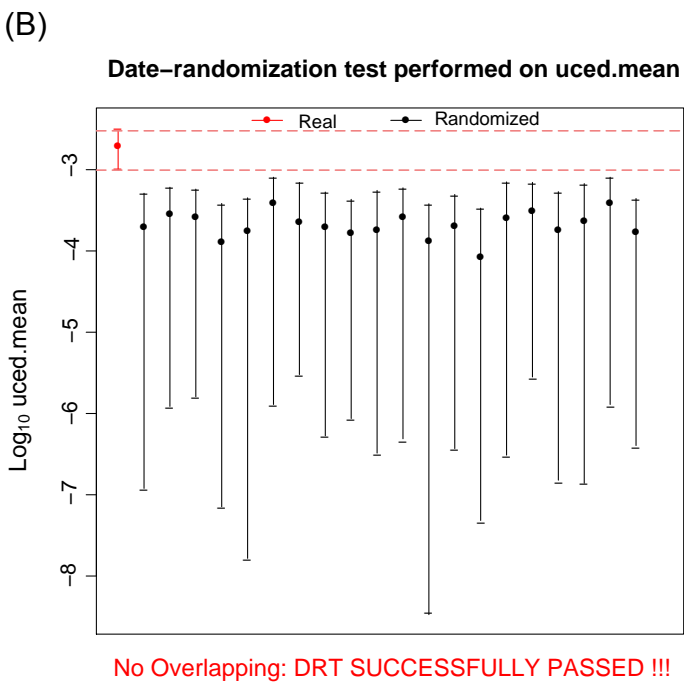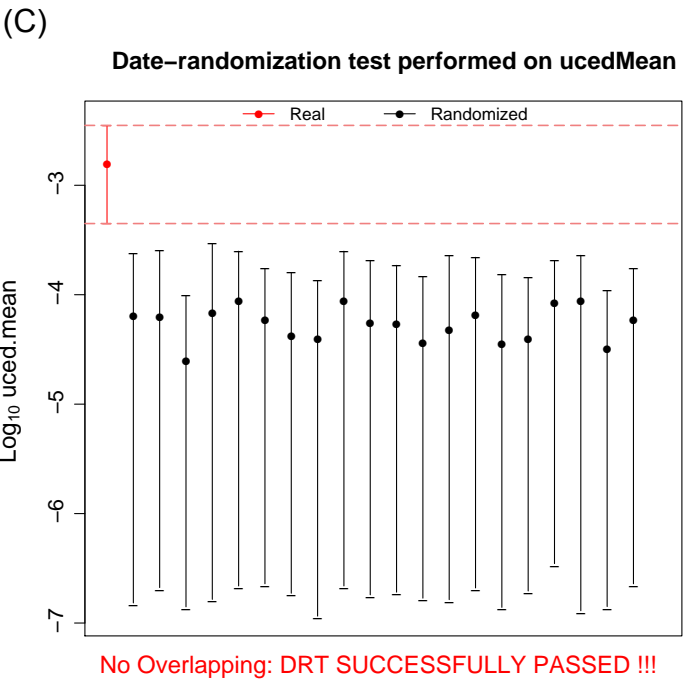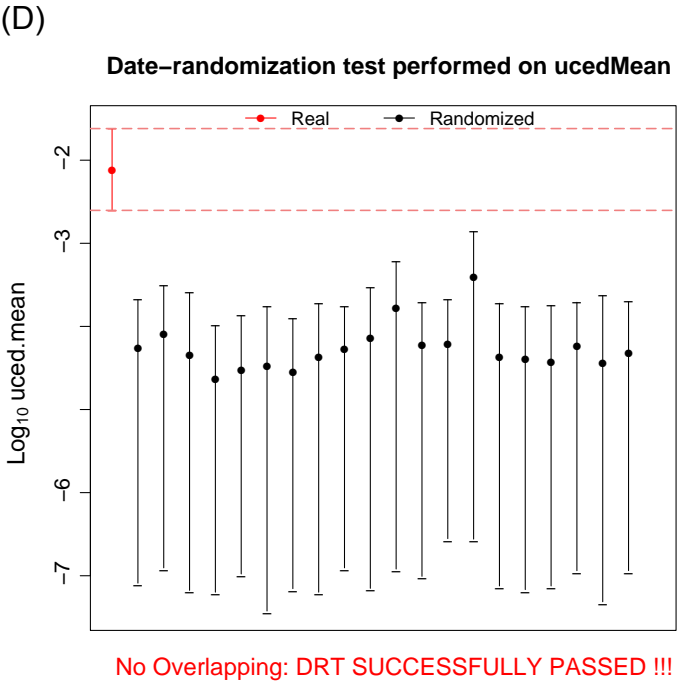

Supplement: Supplementary file 2 [file EVA-10-379-s002.pdf]
