# Supplementary material for: Adaptive evolution and demographic history contribute to the divergent population genetic structure of Potato virus Y between China and Japan
Source: Evol Appl. 2017 Mar 2;10(4):379–90. doi: 10.1111/eva.12459 (PMC5367074; doi:10.1111/eva.12459)
Supplement: Supplementary file 3 [file EVA-10-379-s003.pdf]

Table S1 PVY isolates in this study

Isolate	Country	Host	Date	Accession Number		Reference
				P1	CP	
SL053	China: Guizhou	*Solanum tuberosum*	2005	n/a	EF063710	Zhang & Liu, unpublished
FX24	China: Shandong	*Solanum tuberosum*	2005	n/a	EF592514	Yu et al, unpublished
Fanzhen6	China: Shandong	*Solanum tuberosum*	2005	n/a	EF592515	Yu et al, unpublished
Laiwu3	China: Shandong	*Solanum tuberosum*	2005	n/a	EF592516	Yu et al, unpublished
Fanzhen8	China: Shandong	*Solanum tuberosum*	2005	n/a	EF592521	Liu, et al, unpublished
Laiwu1	China: Shandong	*Solanum tuberosum*	2005	n/a	EF592525	Liu, et al, unpublished
Laiwu9	China: Shandong	*Solanum tuberosum*	2005	n/a	EF592526	Liu, et al, unpublished
Zhengzhou	China: Henan	*Solanum tuberosum*	2007	n/a	JX872404	Liu, et al, unpublished
Lushi	China: Henan	*Solanum tuberosum*	2008	FJ423028	n/a	Liu, et al, unpublished
Guiyang	China: Guizhou	*Solanum tuberosum*	2008	FJ423029	n/a	Liu, et al, unpublished
Caohai	China: Henan	*Solanum tuberosum*	2008	FJ423030	n/a	Liu, et al, unpublished
keshan	China: Heilongjiang	*Solanum tuberosum*	2008	FJ766536	n/a	Liu, et al, unpublished
Minquan	China: Henan	*Solanum tuberosum*	2008	FJ986300	n/a	Liu, et al, unpublished
Heilongjiang	China: Heilongjiang	*Solanum tuberosum*	2008	GU074001	n/a	Liu, et al, unpublished
Dalian	China: Liaoning	*Solanum tuberosum*	2008	GU074002	n/a	Liu, et al, unpublished
HN1	China: Hunan	*Solanum tuberosum*	2007	HQ631374	n/a	Hu et al, 2009
HeB12	China: Hebei	*Solanum tuberosum*	2012	KF771013	KC282358	This study
HeB7	China: Hebei	*Solanum tuberosum*	2012	KF771012	KC282359	This study
HeB5	China: Hebei	*Solanum tuberosum*	2012	KF771010	KC282360	This study
HeB6	China: Hebei	*Solanum tuberosum*	2012	KF771011	KC282361	This study
HeB2	China: Hebei	*Solanum tuberosum*	2012	KF771008	KC282362	This study
HeB15	China: Hebei	*Solanum tuberosum*	2012	KF771014	KC282363	This study
HeB26	China: Hebei	*Solanum tuberosum*	2012	KF771019	KC282364	This study
HeB28	China: Hebei	*Solanum tuberosum*	2012	KF771020	KC282365	This study
HeB19	China: Hebei	*Solanum tuberosum*	2012	KF771016	KC282366	This study

HeB17	China: Hebei	*Solanum tuberosum*	2012	KF771015	KC282367	This study
HeB23	China: Hebei	*Solanum tuberosum*	2012	KF771018	KC282369	This study
HeB4	China: Hebei	*Solanum tuberosum*	2012	KF771009	KC282370	This study
FQ04	China: Fujian	*Solanum tuberosum*	2011	n/a	KC296803	This study
FQ06	China: Fujian	*Solanum tuberosum*	2011	KF722798	KC296806	This study
FQ07	China: Fujian	*Solanum tuberosum*	2011	n/a	KC296815	This study
LH02	China: Fujian	*Solanum tuberosum*	2011	KF722799	KC296828	This study
LH05	China: Fujian	*Solanum tuberosum*	2011	KF722800	KC296826	Shi et al, 2014
LH09	China: Fujian	*Solanum tuberosum*	2011	KF722801	KC296790	This study
LH12	China: Fujian	*Solanum tuberosum*	2011	n/a	KC296789	This study
LH14	China: Fujian	*Solanum tuberosum*	2011	KF722802	KC296816	Shi et al, 2014
LH20	China: Fujian	*Solanum tuberosum*	2011	n/a	KC296818	This study
LH21	China: Fujian	*Solanum tuberosum*	2011	KF722803	KC296791	Shi et al, 2014
LH24	China: Fujian	*Solanum tuberosum*	2011	KF722804	n/a	This study
LY06	China: Fujian	*Solanum tuberosum*	2011	KF722805	KC296823	This study
LY08	China: Fujian	*Solanum tuberosum*	2011	KF722806	KC296793	Shi et al, 2014
LY09	China: Fujian	*Solanum tuberosum*	2011	n/a	KC296796	This study
LY12	China: Fujian	*Solanum tuberosum*	2011	KF722807	KC296794	This study
LY21	China: Fujian	*Solanum tuberosum*	2011	KF722808	KC296798	This study
LY30	China: Fujian	*Solanum tuberosum*	2011	KF722809	KC296800	Shi et al, 2014
LY31	China: Fujian	*Solanum tuberosum*	2011	KF722810	KC296792	This study
LY34	China: Fujian	*Solanum tuberosum*	2011	n/a	KC296821	This study
LY35	China: Fujian	*Solanum tuberosum*	2011	KF722811	n/a	Shi et al, 2014
QK09	China: Fujian	*Solanum tuberosum*	2011	KF722812	KC296824	This study
QK43	China: Fujian	*Solanum tuberosum*	2011	KF722813	n/a	This study
QK44	China: Fujian	*Solanum tuberosum*	2011	KF722814	KC296817	Shi et al, 2014
QK45	China: Fujian	*Solanum tuberosum*	2011	KF722815	KC296827	This study
QK47	China: Fujian	*Solanum tuberosum*	2011	KF722816	KC296810	This study
XQ02	China: Fujian	*Solanum tuberosum*	2011	KF722817	KC296822	This study

XQ03	China: Fujian	*Solanum tuberosum*	2011	KF722818	KC296801	This study
XQ04	China: Fujian	*Solanum tuberosum*	2011	KF722819	KC296802	Shi et al, 2014
XQ05	China: Fujian	*Solanum tuberosum*	2012	KF722820	KC296825	This study
XQ08	China: Fujian	*Solanum tuberosum*	2012	KF722821	KC296797	This study
XQ09	China: Fujian	*Solanum tuberosum*	2012	KF722822	KC296799	This study
XQ10	China: Fujian	*Solanum tuberosum*	2012	KF722823	KC296811	This study
XQ11	China: Fujian	*Solanum tuberosum*	2012	KF722824	KC296804	This study
XT01	China: Fujian	*Solanum tuberosum*	2012	KF722825	KC296830	This study
XT02	China: Fujian	*Solanum tuberosum*	2012	KF722826	KC296820	Shi et al, 2014
XT03	China: Fujian	*Solanum tuberosum*	2012	KF722827	KC296831	This study
XT04	China: Fujian	*Solanum tuberosum*	2012	KF722828	KC296834	Shi et al, 2014
XT05	China: Fujian	*Solanum tuberosum*	2012	KF722829	KC296819	This study
XT07	China: Fujian	*Solanum tuberosum*	2012	KF722830	KC296814	This study
XT08	China: Fujian	*Solanum tuberosum*	2012	KF722831	KC296808	Shi et al, 2014
ZL01	China: Fujian	*Solanum tuberosum*	2012	KF722832	KC296807	This study
ZL02	China: Fujian	*Solanum tuberosum*	2012	KF722833	KC296795	Shi et al, 2014
ZL05	China: Fujian	*Solanum tuberosum*	2012	n/a	KC296832	This study
ZL06	China: Fujian	*Solanum tuberosum*	2012	KF722834	KC296805	This study
ZL07	China: Fujian	*Solanum tuberosum*	2012	KF722835	KC296833	This study
ZL08	China: Fujian	*Solanum tuberosum*	2012	KF722836	KC296813	This study
ZL09	China: Fujian	*Solanum tuberosum*	2012	KF722837	KC296835	This study
ZL10	China: Fujian	*Solanum tuberosum*	2012	KF722838	KC296809	This study
ZL11	China: Fujian	*Solanum tuberosum*	2012	KF722839	n/a	This study
ZL12	China: Fujian	*Solanum tuberosum*	2012	n/a	KC296829	This study
ZL13	China: Fujian	*Solanum tuberosum*	2012	KF722840	n/a	This study
ZL14	China: Fujian	*Solanum tuberosum*	2012	KF722841	KC296812	This study
CD16	China: Hunan	*Solanum tuberosum*	2012	KX451323	KC296836	This study
CD28	China: Hunan	*Solanum tuberosum*	2012	KX451324	KC296837	This study
CD34	China: Hunan	*Solanum tuberosum*	2012	n/a	KC296838	This study

CD39	China: Hunan	*Solanum tuberosum*	2012	KX451325	KC296839	This study
CD45	China: Hunan	*Solanum tuberosum*	2012	KX451326	KC296840	This study
CS1	China: Hunan	*Solanum tuberosum*	2011	KX451327	KC296841	This study
CS10	China: Hunan	*Solanum tuberosum*	2011	KX451328	KC296842	This study
CS11	China: Hunan	*Solanum tuberosum*	2011	KX451329	KC296843	This study
CS12	China: Hunan	*Solanum tuberosum*	2011	KX451330	KC296844	This study
CS13	China: Hunan	*Solanum tuberosum*	2011	KX451331	KC296845	This study
CS14	China: Hunan	*Solanum tuberosum*	2011	KX451332	KC296846	This study
CS15	China: Hunan	*Solanum tuberosum*	2011	KX451333	KC296847	This study
CS16	China: Hunan	*Solanum tuberosum*	2011	KX451334	KC296848	This study
CS17	China: Hunan	*Solanum tuberosum*	2011	KX451335	KC296849	This study
CS18	China: Hunan	*Solanum tuberosum*	2011	KX451336	KC296850	This study
CS19	China: Hunan	*Solanum tuberosum*	2011	KX451337	KC296851	This study
CS2	China: Hunan	*Solanum tuberosum*	2011	KX451338	KC296852	This study
CS20	China: Hunan	*Solanum tuberosum*	2011	KX451339	KC296853	This study
CS21	China: Hunan	*Solanum tuberosum*	2011	KX451340	KC296854	This study
CS22	China: Hunan	*Solanum tuberosum*	2011	KX451341	KC296855	This study
CS23	China: Hunan	*Solanum tuberosum*	2011	KX451342	KC296856	This study
CS24	China: Hunan	*Solanum tuberosum*	2011	KX451343	n/a	This study
CS26	China: Hunan	*Solanum tuberosum*	2011	n/a	KC296857	This study
CS27	China: Hunan	*Solanum tuberosum*	2011	n/a	KC296858	This study
CS28	China: Hunan	*Solanum tuberosum*	2011	KX451344	KC296859	This study
CS29	China: Hunan	*Solanum tuberosum*	2011	KX451345	n/a	This study
CS30	China: Hunan	*Solanum tuberosum*	2011	n/a	KC296861	This study
CS31	China: Hunan	*Solanum tuberosum*	2011	KX451346	KC296862	This study
CS36	China: Hunan	*Solanum tuberosum*	2011	KX451347	KC296863	This study
CS37	China: Hunan	*Solanum tuberosum*	2011	KX451348	KC296864	This study
CS4	China: Hunan	*Solanum tuberosum*	2011	KX451349	KC296865	This study
CS5	China: Hunan	*Solanum tuberosum*	2011	KX451350	KC296866	This study

CS9	China: Hunan	*Solanum tuberosum*	2011	KX451351	n/a	This study
GZ2	China: Guizhou	*Solanum tuberosum*	2011	n/a	KC506388	Gao et al, 2013
HeN12	China: Henan	*Solanum tuberosum*	2012	n/a	KC506389	Gao et al, 2013
SD1	China: Shandong	*Solanum tuberosum*	2012	n/a	KC506394	Gao et al, 2013
GF_YL20	China: Shaanxi	*Solanum tuberosum*	2011	KJ634023	KJ634023	Gao et al, 2014
ShX14	China: Shaanxi	*Solanum tuberosum*	2011	KJ634024	KJ634024	Gao et al, 2015
CF_YL21	China: Shaanxi	*Solanum tuberosum*	2011	KJ801915	KJ801915	Chang et al, 2015
HN2	China: Hunan	*Solanum tuberosum*	2007	GQ200836	GQ200836	Hu et al, 2009
NTND6	Japan: Kyushu	*Solanum tuberosum*	1995–2000	AB331515	AB331515	Ogawa T, et al, 2008
NTNOK105	Japan: Okinawa	*Solanum tuberosum*	1995–2000	AB331516	AB331516	Ogawa T, et al, 2008
NTNHO90	Japan: Hokkaido	*Solanum tuberosum*	1995–2000	AB331517	AB331517	Ogawa T, et al, 2008
NTNNN99	Japan: Honshu	*Solanum tuberosum*	1995–2000	AB331518	AB331518	Ogawa T, et al, 2008
NTNON92	Japan: Kyushu	*Solanum tuberosum*	1995–2000	AB331519	AB331519	Ogawa T, et al, 2008
NTNHIR3	Japan: Honshu	*Solanum tuberosum*	2012	AB711143	AB711143	Ogawa T, et al, 2012
NTNKGAM1	Japan: Kyushu	*Solanum tuberosum*	2012	AB711144	AB711144	Ogawa T, et al, 2012
NTNKGAM2	Japan: Kyushu	*Solanum tuberosum*	2012	AB711145	AB711145	Ogawa T, et al, 2012
NTNTK1	Japan: Honshu	*Solanum tuberosum*	2012	AB711146	AB711146	Ogawa T, et al, 2012
OAO21	Japan: Honshu	*Solanum tuberosum*	2012	AB711147	AB711147	Ogawa T, et al, 2012
OAO22	Japan: Honshu	*Solanum tuberosum*	2012	AB711148	AB711148	Ogawa T, et al, 2012
OHO37	Japan: Hokkaido	*Solanum tuberosum*	2012	AB711149	AB711149	Ogawa T, et al, 2012
OKUM4	Japan: Kyushu	*Solanum tuberosum*	2012	AB711150	AB711150	Ogawa T, et al, 2012
ONGI3	Japan: Kyushu	*Solanum tuberosum*	2012	AB711151	AB711151	Ogawa T, et al, 2012
ONGOB6	Japan: Kyushu	*Solanum tuberosum*	2012	AB711152	AB711152	Ogawa T, et al, 2012
OH	Japan: Hokkaido	*Solanum tuberosum*	2012	AB714134	AB714134	Ogawa T, et al, 2012
T13	Japan: Hokkaido	*Solanum tuberosum*	1989	AB714135	AB714135	Ogawa T, et al, 2012
NTN68	Japan: Kyushu	*Solanum tuberosum*	1995–2000	AB331526	AB331541	Ogawa T, et al, 2008
NTND7	Japan: Kyushu	*Solanum tuberosum*	1995–2000	AB331521	AB042811	Ogawa T, et al, 2008
NTNDG44	Japan: Kyushu	*Solanum tuberosum*	1995–2000	AB331529	AB331543	Ogawa T, et al, 2008
NTNHO91	Japan: Hokkaido	*Solanum tuberosum*	1995–2000	AB331535	AB331548	Ogawa T, et al, 2008

NTNHO92	Japan: Hokkaido	*Solanum tuberosum*	1995–2000	AB331536	AB331549	Ogawa T, et al, 2008
NTNHO95	Japan: Hokkaido	*Solanum tuberosum*	1995–2000	AB331537	AB331550	Ogawa T, et al, 2008
NTNK	Japan: Kyushu	*Solanum tuberosum*	1995–2000	AB331528	AB025417	Ogawa T, et al, 2008
NTNK110	Japan: Kyushu	*Solanum tuberosum*	1995–2000	AB331523	AB331538	Ogawa T, et al, 2008
NTNK111	Japan: Kyushu	*Solanum tuberosum*	1995–2000	AB331524	AB331539	Ogawa T, et al, 2008
NTNK114	Japan: Kyushu	*Solanum tuberosum*	1995–2000	AB331525	AB331540	Ogawa T, et al, 2008
NTNN2	Japan: Kyushu	*Solanum tuberosum*	1995–2000	AB331520	AB025415	Ogawa T, et al, 2008
NTNN14	Japan: Kyushu	*Solanum tuberosum*	1995–2000	AB331522	AB042812	Ogawa T, et al, 2008
NTNNNO	Japan: Honshu	*Solanum tuberosum*	1995–2000	AB331532	AB331545	Ogawa T, et al, 2008
NTNOK102	Japan: Okinawa	*Solanum tuberosum*	1995–2000	AB331533	AB331546	Ogawa T, et al, 2008
NTNOK104	Japan: Okinawa	*Solanum tuberosum*	1995–2000	AB331534	AB331547	Ogawa T, et al, 2008
NTNT	Japan: Kyushu	*Solanum tuberosum*	1995–2000	AB331527	AB331542	Ogawa T, et al, 2008
NTNUG70	Japan: Kyushu	*Solanum tuberosum*	1995–2000	AB331530	AB042813	Ogawa T, et al, 2008

Literature cited:

Chang F, Gao F, Shen J, Zou W, Zhao S, Zhan J. 2015. Complete genome analysis of a PVY$^{N\text{-}Wi}$ recombinant isolate from *Solanum tuberosum* in China. *Potato research,* **58**:377-389

Gao F, Shen J, Shi F, Fang Z, Xie L, Zhan J. 2013. Detection and molecular variation of Potato virus Y CP gene in China. *Scientia agricultura Sinica,* **46**:3125-3133

Gao, F., Chang, F., Shen, J., Shi, F., Xie, L. and Zhan, J. 2014. Complete genome analysis of a novel recombinant isolate of potato virus Y from China. *Archives of virology,* **159,** 3439-3442.

Gao F, Chang F, Shen J, Xie L, Zhan J. 2015. Complete genome analysis of a PVY$^{NTN\text{-}NW}$ recombinant isolate from Yulin of China. *Scientia agricultura Sinica,* **48**:270-279

Hu X, He C, Xiao Y, Xiong X, Nie X. 2009. Molecular characterization and detection of recombinant isolates of *potato virus Y* from China. *Archives of virology* **154**:1303-1312

Ogawa, T., Nakagawa, A., Hataya, T. and Ohshima, K. 2012. The genetic structure of populations of *Potato virus Y* in Japan; based on the analysis of 20 full genomic sequences. *Journal of Phytopathology,* **160,** 661-673.

Ogawa, T., Tomitaka, Y., Nakagawa, A. and Ohshima, K. 2008. Genetic structure of a population of *Potato virus Y* inducing potato tuber necrotic ringspot disease in Japan; comparison with North American and European populations. *Virus research,* **131,** 199-212.

Shi F, Gao F, Shen J, Chang F, Zhan J. 2014. Sequence variation of P1 gene in *Potato virus Y* isolated from Fujian province. *Herditas*, **36**: 713-722.
