# Supplementary material for: Adaptive evolution and demographic history contribute to the divergent population genetic structure of Potato virus Y between China and Japan
Source: Evol Appl. 2017 Mar 2;10(4):379–90. doi: 10.1111/eva.12459 (PMC5367074; doi:10.1111/eva.12459)
Supplement: Supplementary file 4 [file EVA-10-379-s004.pdf]

Table S2. List of sequences with recombinant backgrounds detected in the PVY P1 and CP genes by RDP Suites

Gene	Recombinant	Break point (nt)[a]	*P*-value determined using seven different programs						
			RDP	Geneconv	BootScan	Maxchi	Chimarera	SiScan	3Seq
P1	Lushi, Dalian, FQ06, LH02, LH05, LH24, LY06, LY12, LY21, QK09, QK43, QK44, QK45, QK47, XQ02, XQ10, XT02, XT03, XT05, XT07, XT08, ZL01, ZL06, ZL07, ZL08, ZL10, ZL13, ZL14, HeB2, HeB4, HeB6, HeB15, CF, CD16, CD39, CS10, CS2, CS22, CS29, CS37, CS9	568-20	NS	7.74×10^{-25}	8.00×10^{-24}	4.40×10^{-16}	4.07×10^{-15}	1.37×10^{-30}	1.89×10^{-43}
	CS5, Dalian, FQ06, LH02, LH24, LY06, LY12, QK09, QK43, QK44, QK45, QK47, XQ02, XQ10, XT02, XT03, XT05, XT07, XT08, ZL01, ZL06, ZL07, ZL08, ZL10, ZL13, ZL14, HeB2, HeB4, HeB6, HeB15, CF_YL21, CD16, CD39, CS10, CS2, CS22, CS37, CS9	819-310	NS	1.48×10^{-21}	NS	3.85×10^{-17}	3.84×10^{-18}	4.34×10^{-19}	2.34×10^{-39}
	LH05	426-567	4.62×10^{-14}	1.24×10^{-12}	1.27×10^{-02}	2.32×10^{-09}	8.98×10^{-10}	5.76×10^{-12}	7.88×10^{-19}
CP	ZL09	796-310	NS	8.60×10^{-08}	3.19×10^{-06}	1.07×10^{-12}	3.10×10^{-08}	4.18×10^{-14}	5.74×10^{-17}
	CS28	424-31	2.47×10^{-03}	1.57×10^{-02}	NS	5.29×10^{-09}	1.53×10^{-05}	1.69×10^{-02}	4.68×10^{-08}
	Laiwu9, CS10, CS13, CS14, CS26, CS27, NTNHIR3, NTNKGAM2, NTNTK1, T13,	766-568	NS	3.14×10^{-08}	1.51×10^{-06}	3.92×10^{-07}	2.51×10^{-06}	2.86×10^{-08}	3.04×10^{-10}

[a], the break points listed refer to their position in the alignment;

NS, No significant P-value was recorded for this recombination event using this method.
