# Supplementary material for: Adaptive evolution and demographic history contribute to the divergent population genetic structure of Potato virus Y between China and Japan
Source: Evol Appl. 2017 Mar 2;10(4):379–90. doi: 10.1111/eva.12459 (PMC5367074; doi:10.1111/eva.12459)
Supplement: Supplementary file 5 [file EVA-10-379-s005.pdf]

Table S3. Haplotype diversity assessed by bootstrap resampling with 1000 replicates

Gene	Country	Haplotype diversity	95% Confidence intervals	P-value
P1	China	0.988	0.970 - 0.998	0.990^{ns}
	Japan	0.989	0.972 - 0.998	0.098^{ns}
CP	China	0.967	0.924 - 0.989	1.000^{ns}
	Japan	0.972	0.936 - 0.991	0.050*

^{ns}, not significant; *, 0.01< *P-value* <0.05; **, 0.001< *P-value* <0.01; ***, *P-value* <0.001
